# Effect of CrF_3_ Addition on Photoluminescence Properties of Lead-Free Cs_4_SnBr_6−x_F_x_ Zero-Dimensional Perovskite

**DOI:** 10.3390/ma16186309

**Published:** 2023-09-20

**Authors:** Jianni Chen, Haixia Wu, Yaqian Huang, Jisheng Xu, Xinye Lu, Wendi Zhou, Jie Song, Rui Huang

**Affiliations:** School of Materials Science and Engineering, Hanshan Normal University, Chaozhou 521041, China

**Keywords:** photoluminescence, self-trapped exciton, Cs_4_SnBr_6_, CrF_3_

## Abstract

Zero-dimensional (0D) tin halide perovskites, characterized by their broadband and adjustable emissions, high photoluminescence quantum yield, and absence of self-absorption, are crucial for the fabrication of high-efficiency optoelectronic devices, such as LEDs, solar cells, and sensors. Despite these attributes, boosting their emission efficiency and stability poses a significant challenge. In this work, Cr^3+^-doped Cs_4_SnBr_6−x_F_x_ perovskites were synthesized using a water-assisted wet ball-milling method. The effect of CrF_3_ addition on photoluminescence properties of Cs_4_SnBr_6−x_F_x_ Perovskites was investigated. We found that Cr^3+^-doped Cs_4_SnBr_6−x_F_x_ Perovskites exhibit a broad emission band, a substantial Stokes shift, and an efficient green light emission centered at about 525 nm at ambient temperature. The derived photoluminescence quantum yield amounted to as high as 56.3%. In addition, these Cr^3+^-doped Cs_4_SnBr_6−x_F_x_ perovskites outperform their undoped counterparts in terms of thermal stability. Through a comprehensive analysis of photoluminescence measurements, our findings suggested that the elevated photoluminescence quantum yield can be attributed to the enhanced exciton binding energy of self-trapped excitons (STEs) and the suitable electron−phonon coupling resulting from the substantial distortion of [SnBr_6_]^4−^ octahedra instigated by the addition of CrF_3_.

## 1. Introduction

In the past decade, metal halide perovskites, characterized by their unique photophysical properties, including high photoluminescence quantum yield (PL QY) and variable light emission, have garnered significant attention [1,2,3]. Among the various alternatives, all-inorganic perovskites, especially inorganic CsPbX_3_ (X = Cl, Br, I) nanocrystals (NCs), have attracted more attention than others because of their impressive diffusion length, efficient PL QY, and superior stability compared to organic–inorganic hybrid perovskites [4,5,6]. Furthermore, they exhibit a high tolerance to surface defects [7]. As such, directly synthesized CsPbBr_3_ NCs can achieve PL QY as high as 90% even without specific surface modifications [8]. Nevertheless, the pronounced toxicity of lead in CsPbX_3_ presents a significant challenge, carrying implications for both human health and environmental ecology, thus limiting their practical application. Given its ns^2^ electronic configuration, electronegativity, and ionic radii, Sn^2+^ shares considerable similarities with Pb^2+^. Consequently, its compounds, which present a lower toxicity profile, are viewed as promising alternatives to the Pb^2+^-containing perovskites [9,10]. Sn^2+^ compounds are generally considered more promising alternatives to Pb^2+^-based perovskites owing to their lower toxicity. However, Sn-based perovskites bear an inherently lower formation energy for defects (~250 meV), which facilitates the creation of a substantial defect density of up to 10^19^ cm^–3^, thereby reducing the PL QY of these perovskites [9]. So far, substantial efforts have been directed towards the study of all-inorganic Sn-based perovskites in an attempt to understand the innate relationship among crystal structure, photophysical mechanism, and photoelectric conversion properties. For instance, Wang et al. presented a versatile colloidal synthetic method for CsSnX_3_ perovskite nanocrystals (NCs), using a shrewd blend of cost-effective surfactants, such as oleic acid (OA), oleylamine (OAm), and phosphatidylcholine. This method resulted in the production of colloidal CsSnI_3_, CsSnI_2.5_Br_0.5_, and CsSnI_2.25_Br_0.75_ perovskite NCs, boasting PL QYs of 12.0%, 10.9%, and 9.5%, respectively [11]. Similarly, Liu et al. crafted a colloidal synthesis strategy enabling comprehensive fine-tuning of the reactant ratio that still culminated in the formation of CsSnI_3_ NCs. Their approach successfully yielded narrow-band-emissive CsSnI_3_ NCs with a record-breaking emission quantum yield of 18.4%, achieved by reducing the density of tin vacancies (V_Sn_) in the NCs lattice [10]. Despite these advancements in CsSnX_3_ perovskites, the luminescent efficiency remains insufficient for practical applications. Therefore, further exploration and improvement are necessary to fulfill the demands of real-world applications.

In recent years, there has been a surge of attention towards zero-dimensional (0D) tin halide perovskites (Cs_4_SnX_6_, X = Br and I) with self-trapping excitons (STEs) emission, primarily due to their remarkable PL QY. These materials are characterized by the presence of luminescent [SnX_6_]^−4^ tin halide octahedra, which are bound by Cs cations. Up to now, a wealth of research has delved into the fascinating properties of 0D tin halide perovskites, showcasing their potential for optoelectronic applications [12,13]. For instance, Kovalenko et al. revealed efficient green-yellow emission sourced from self-trapped excitons in Cs_4_SnX_6_, boasting an admirable PL QY of 15 ± 5% at room temperature [12]. Similarly, Quan’s group succeeded in synthesizing high-quality Cs_4_SnX_6_ (X = Br, I) nanocrystals of unique shapes and narrow size distributions, achieving an impressive PL QY of up to 21% for these Cs_4_SnX_6_ nanocrystals [13]. To address the air instability challenge of Cs_4_SnX_6_, primarily linked to the oxidation of Sn^2+^ to Sn^4+^, Zhang and his colleagues introduced an innovative approach. They replaced SnBr_2_, susceptible to easy oxidation, with SnF_2_ as the tin source. Their approach notably bolstered the structural stability of Cs_4_SnX_6_ perovskite by leveraging fluorine’s ability to suppress Sn^2+^ oxidation effectively [14]. While 0D tin halide perovskites demonstrate remarkable PL performance, the journey towards their commercialization calls for dedicated efforts to boost both their PL efficiency and stability. Contrasting with luminescence in metal halide perovskite QDs, the formation of STEs in 0D tin halide perovskites demands lattice distortion, accompanied by strong electron–phonon coupling. This electron–phonon coupling, a consequence of the soft lattice, intensifies the probability of STEs, thereby promoting increased STEs emission [15,16]. Prior studies have indicated that lattice distortion in 0D metal halide perovskites can be modulated by adjusting factors such as chemical composition, temperature, and pressure [15,16,17,18]. This fine-tuning enables meticulous control over the STE state, optimizing luminescence performance in turn. For example, Ma and his collaborators reported that, in Cs_4_PbBr_6_ nanocrystals, under ambient temperature conditions, an emission induced by pressure is observed upon reaching a threshold of 3.01 GPa. This heightened emission under pressure is attributed to an enhancement in optical activity and a surge in the binding energy of STEs in the high-pressure phase. This is conjectured to stem from a marked distortion and increased rigidity in the [PbBr_6_]^4−^ octahedra under compressive stress [17]. In our preceding research, we discovered that Mn^2+^ doping into Cs_4_SnX_6_ perovskites not only broadens the emission spectrum, but also amplifies the distortion of [SnX_6_]^4−^ octahedra, strengthening the electron–phonon coupling in the process. This sequence of events enhances the density of STE states, resulting in boosted luminescence efficiency [18].

In this work, Cr^3+^-doped Cs_4_SnBr_6−x_F_x_ Perovskites were synthesized by a water-assisted wet ball-milling method. The effect of Cr^3+^ doping on photoluminescence properties of Cs_4_SnBr_6−x_F_x_ Perovskites was investigated. It is found that Cr^3+^-doped Cs_4_SnBr_6−x_F_x_ perovskites exhibit a broad emission band, a substantial Stokes shift, and efficient green light emission centered at about 525 nm at ambient temperature. The derived photoluminescence quantum yield amounted to as high as 56.3%. In addition, these Cr^3+^-doped Cs_4_SnBr_6−x_F_x_ perovskites outperform their undoped counterparts in terms of thermal stability. Based on the comprehensive analysis of photoluminescence measurements, the enhancement in PL is discussed in terms of the binding energies of STEs and electron–phonon coupling instigated by the addition of CrF_3_.

## 2. Materials and Methods

**Figure 1:**
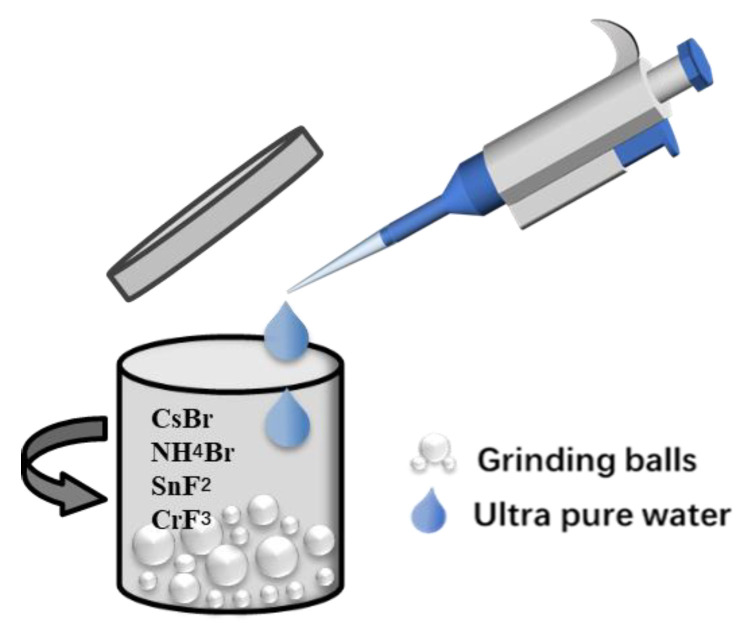
Illustration of the procedure for fabricating Cr^3+^-doped Cs_4_SnBr_6_.

Cs_4_SnBr_6−x_F_x_ samples were procured through a water-assisted wet ball-milling procedure. The reactant precursors employed were cesium bromide (4 mmol, CsBr, Aladdin, 99.9%), stannous fluoride (1 mmol, SnF2, Macklin, 99.9%), and ammonium bromide (1 mmol, NH_4_Br, Aladdin, 99.99%). To derive Cr^3+^-doped Cs_4_SnBr_6−x_F_x_ with varying concentrations, the molar ratios of CsBr, SnF_2_, and NH_4_Br were retained at 4 mmol, 1 mmol, and 2 mmol, respectively, while the molar ratio of CrF_3_ was set at 0 mmol, 0.05 mmol, 0.1 mmol, 0.3 mmol, and 0.5 mmol, correspondingly. The precursor blend was initially placed in a jar and amalgamated with 60 μL of deionized water, followed by ball milling for 30 min at 600 rpm. The material was then dried in a vacuum oven for 60 min at 60 °C. After cooling to ambient temperature, the Cr^3+^-doped Cs_4_SnBr_6−x_F_x_ powder was procured by conducting ball milling for an additional 30 min at 600 rpm. Figure 1 depicts the synthesis process of Cr^3+^-doped Cs_4_SnBr_6−x_F_x_ through water-assisted ball-milling at room temperature. For PL measurement, the power samples were carefully transferred into the well of the FLS1000 solid sample holder (Livingstone, UK). The PL characteristics were evaluated using an Edinburgh Instrument FLS1000 PL spectrometer (Livingstone, UK). The PL was excited at an angle of 30 degrees relative to the surface of the samples and was collected at an angle of 60 degrees to the surface. Time-resolved PL spectra and PL excitation (PLE) spectra were also documented utilizing the same instrument. The crystallographic structures of Cs_4_SnBr_6−x_F_x_ were analyzed via X-ray diffraction (XRD) with a Bruker D8 Advance instrument (Karlsruhe, Germany). XRD examinations were performed at 35 kV and 35 mA to determine the samples’ crystal structure. The compositional analysis of Cs_4_SnBr_6−x_F_x_ was achieved via energy dispersive spectroscopy (EDS) utilizing a Bruker EDS QUANTAX system (Karlsruhe, Germany). The surface morphology and microstructure of Cs4SnBr6 were explored using scanning electron microscopy (SEM) implemented on a Hitachi SU5000 SEM instrument (Tokyo, Japan).

## 3. Results and Discussion

**Figure 2:**
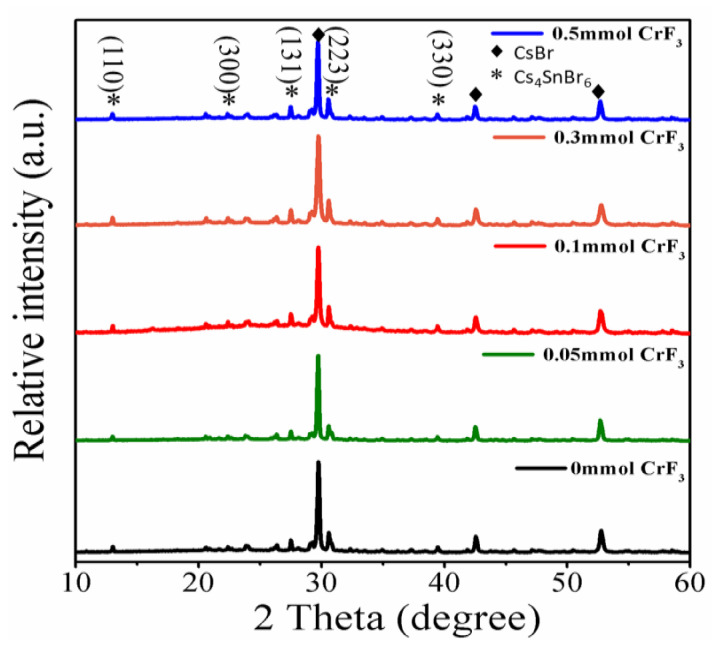
XRD patterns of Cr^3+^-doped samples with different CrF_3_ addition, respectively.

The XRD profiles of the Cr^3+^-doped samples shown in Figure 2 illustrate the simultaneous phase presence of Cs_4_SnBr_6_ and CsBr, indicating an incomplete reaction of the CsBr powder precursors in the solid-state synthesis. Besides the diffraction peaks observed at 29.7°, 42.6°, and 52.7°, ascribed to the CsBr phase, we also identify diffraction peaks corresponding to the (110), (300), (131), (223), and (330) crystal planes of the Cs_4_SnBr_6_ phase. These findings align with those previously reported for SnF_2_-derived Cs_4_SnBr_6_ [14,19]. The clarity of these diffraction peaks intimates high crystallinity in these samples. Consequently, our observations indicate that Cr^3+^ doping does not interfere with the primary crystal structure of Cs_4_SnBr_6_ and is likely incorporated into the host lattice.

**Figure 3:**
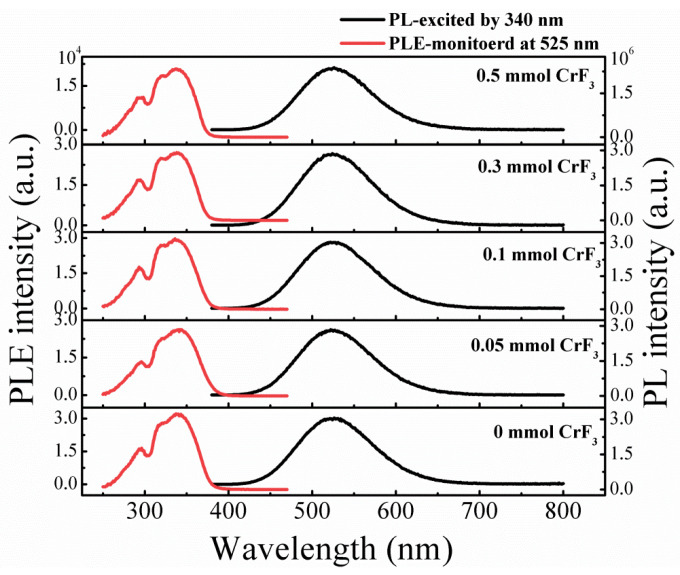
PL and PLE spectra of Cr^3+^-doped samples with different CrF_3_ addition, respectively. The PL spectra are excited by the 340 nm line from Xe lamp. The PLE spectra are monitored at 525 nm.

**Figure 4:**
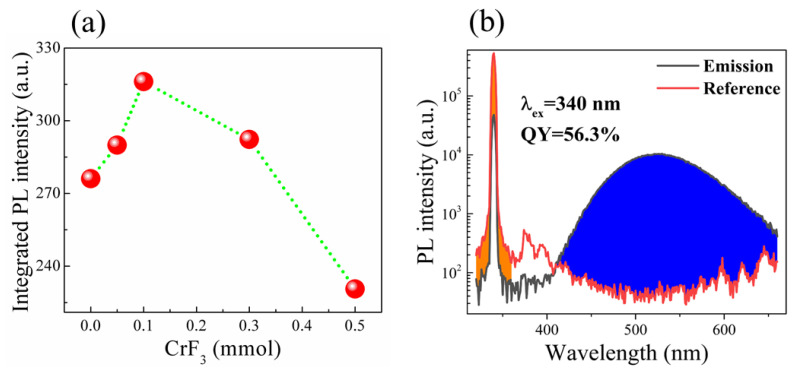
(**a**) PL intensity vs. CrF_3_ addition. Inset is a photo of PL from the oxidized sample illuminated by a 6 W UV lamp. (**b**) The PL QY derived from the excitation and emission spectra for the Cs_4_SnBr_6_ with the molar ratio of CrF_3_ of 0.1 mmol. The PL QYs were directly measured using a PL spectrometer (FLS1000 Edinburgh Instrument) with an integrating sphere and excitation light at 340 nm.

Figure 3 presents the PL spectra of the pure sample alongside those of samples subjected to various CrF_3_ addition. A broad emission, peaking at approximately 525 nm, is observed across all sample types. The emission band, marked by a substantial full width at half maximum of approximately 110 nm, is consistent across all samples. The PLE spectra for all samples, monitored at 525 nm, peak around 340 nm, resulting in a considerable Stokes shift of approximately 1.30 eV, as depicted in Figure 3. The green light emissions are clearly visible to the naked eye in a well-lit room under illumination from a 6 W UV lamp, as displayed in Figure 4a. Notably, the addition of CrF_3_ into the Cs_4_SnBr_6_ perovskites yielded an increase in PL intensity (demonstrated in Figure 4a). This intensity reached a maximal value when the molar ratio of CrF_3_ was elevated to 0.1 mmol, with the PL quantum yield (QY) of 56.3%, as illustrated in Figure 4b and Supporting Information (PL QY calculation). However, the PL intensity of the Cs_4_SnBr_6_ samples experienced a rapid decrease when the CrF_3_ addition was further increased.

**Figure 5:**
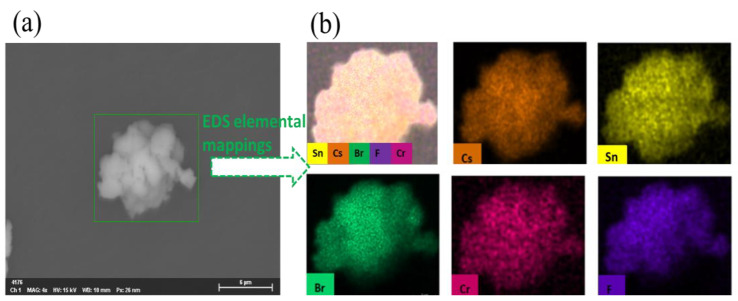
(**a**) SEM image and (**b**) EDS elemental maps of Cs, Sn, Cr, Br, and F for a typical Cr^3+^-doped sample prepared with the CrF_3_ of 0.1 mmol, respectively.

**Figure 6:**
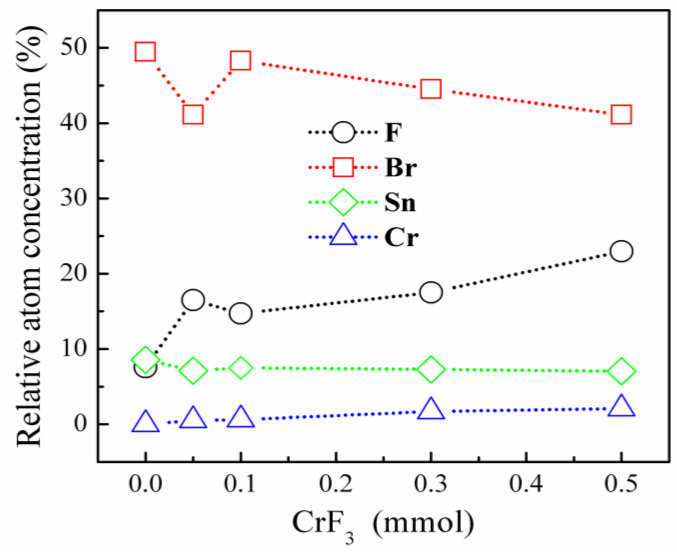
Relative atom concentration of Cs, Sn, Cr, Br, and F in samples as a function of CrF_3_ molar ratio.

Figure 5a depicts the SEM image of the Cr^3+^-doped sample prepared with a molar ratio of 0.1 mmol for CrF_3_. The EDS spectrum confirms the existence of Cs, Sn, Cr, Br, and F elements within the Cs_4_SnBr_6_ structure, which are uniformly distributed as demonstrated in the EDS mappings shown in Figure 5b. Figure 6 presents a gradual increase in the relative atomic concentration of Cr corresponding to an increasing molar ratio of CrF_3_. Conversely, the Sn atomic concentration exhibits a declining trend with an increment in the molar ratio of CrF_3_. These observations strongly indicate the substitution of a greater number of Sn^2+^ ions by the smaller Cr^3+^ ions, aligning with the XRD results presented in Figure 2. This substitution likely results in a substantial distortion of the octahedra within the Cs_4_SnBr_6_, thereby strengthening the electron−phonon coupling. Additionally, Figure 6 reveals a rising relative atomic concentration of F with an increasing molar ratio of CrF_3_, whereas the Br atomic concentration gradually decreases under the same conditions. According to the Hard and Soft Acids and Bases (HSAB) theory, soft acids, such as Sn^2+^, exhibit a preference for coordination with soft bases, like Br^−^, over harder bases such as F^−^. Conversely, hard acids, such as Cr^3+^, have a natural affinity for coordinating with hard bases, favoring F^−^ over softer bases like Br^−^. Therefore, when substituting Cr^3+^ with Sn^2+^, there is a gradual increment in the concentration of the F atom within the sample. The F^−^ ions appear to play a critical role in mitigating the oxidation of Sn^2+^. The high electronegativity and small size of F^−^ make it a particularly effective ligand in stabilizing the Sn^2+^ oxidation state. This mechanism favors the stability of the Cs_4_SnBr_6_ crystal structure.

**Figure 7:**
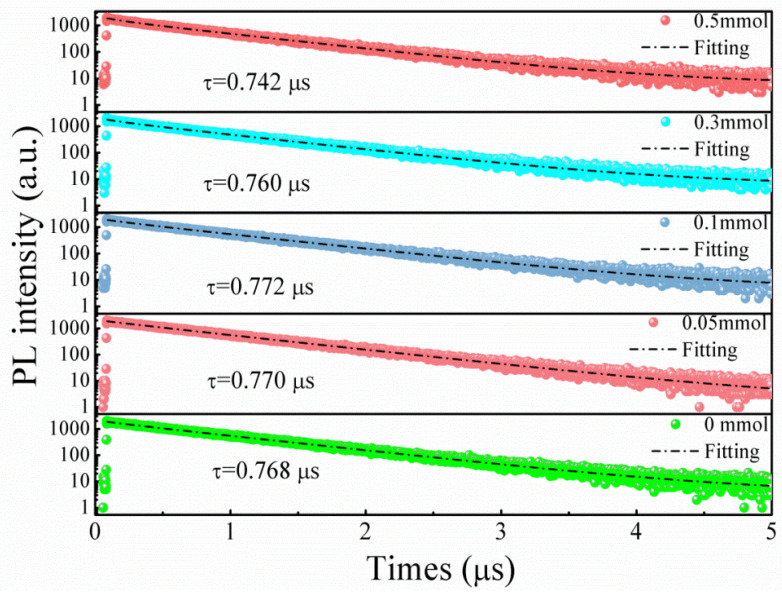
Time-resolved PL decay traces, captured at 530 nm, in Cr^3+^-doped samples with different CrF_3_ additions. Each measurement was carried out at a 375 nm excitation wavelength, utilizing 70 ps laser excitation pulses.

**Figure 8:**
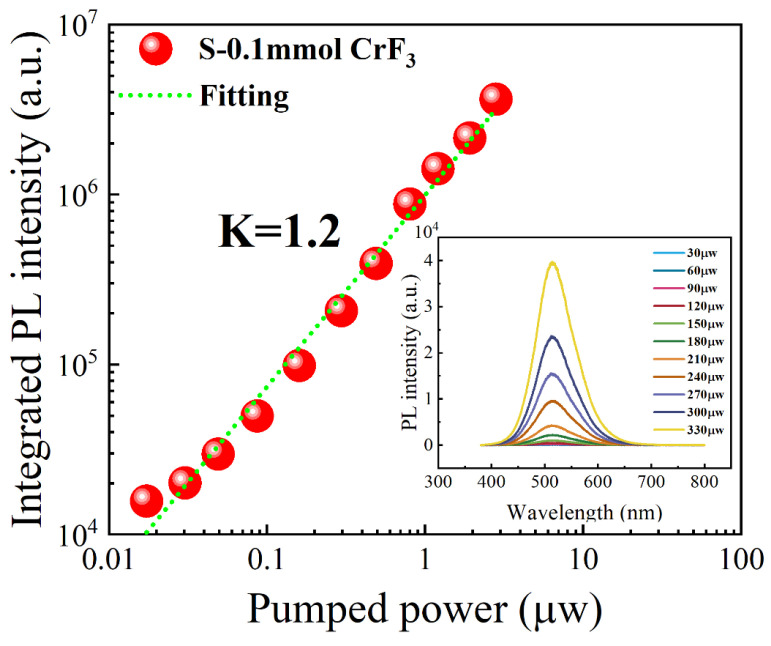
The integrated PL intensity subjected to varied excitation powers for sample doped with a CrF_3_ molar ratio of 0.1 mmol. The solid red lines represent the theoretically fitted curves. The insets display the PL spectra derived from the corresponding samples under different excitation powers.

To understand the PL characteristics, we performed measurements of PL decay curves, using a 375 nm excitation wavelength facilitated by 70 ps laser pulses, as shown in Figure 7. It is found that the PL decay curve linked to the green emission can be appropriately modeled using a biexponential decay function [20]. The intensity-weighted average PL lifetimes were derived, as shown in Figure 7. Notably, all samples, despite the variations in CrF_3_ addition, reveal a green emission with slow decay, exhibiting a considerably long radiative lifetime of around 0.78 μs. These observations suggest that the PL from Cs_4_SnBr_6−x_F_x_ perovskites with varying CrF_3_ addition originates from similar photophysical processes. Figure 8 presents the excitation power dependency of PL for samples doped with a CrF_3_ molar ratio of 0.1 mmol. As the insets of Figure 8 illustrate, an increase in excitation power, from 30 to 330 μW, is paired with a corresponding boost in PL intensity. The PL peak position remains consistent, unfazed by fluctuations in the excitation power. In addition, a linear correlation is observed between the PL intensity and laser power within the 30 to 330 μW range. The PL intensity (I) can be defined via the equation $I={\eta I}_{0}^{k}$, where I_0_ denotes the excitation power, η indicates the PL efficiency, and the exponent k pertains to the radiative recombination process [21]. For excitonic recombination, the value of k falls within the range of 1 to 2. In the case of band-gap emission, which corresponds to electron-hole bimolecular recombination, k equals 2; and k assumes a value less than 1 when transitions involve an impurity or are related to donor–acceptor interactions. By performing a linear fit of ln(I/η) versus ln(I_0_), we can determine the value of k, which in the case of Cs_4_SnBr_6_ doped with a CrF_3_ molar ratio of 0.1 mmol, is 1.2. This finding strongly indicates that the observed green emission stems from exciton recombination [21]. Thus, considering the significant Stokes shift of approximately 1.30 eV, coupled with the wide full-width at half-maximum of the emission band of roughly 110 nm (as presented in Figure 3), along with the extended radiative lifetime of around 0.78 μs (demonstrated in Figure 7), the green emission is presumed to arise from the radiative recombination of STEs, which is prompted by Jahn–Teller distortion of [SnBr_6_]^4−^ octahedra in 0D perovskite [22,23].

**Figure 9:**
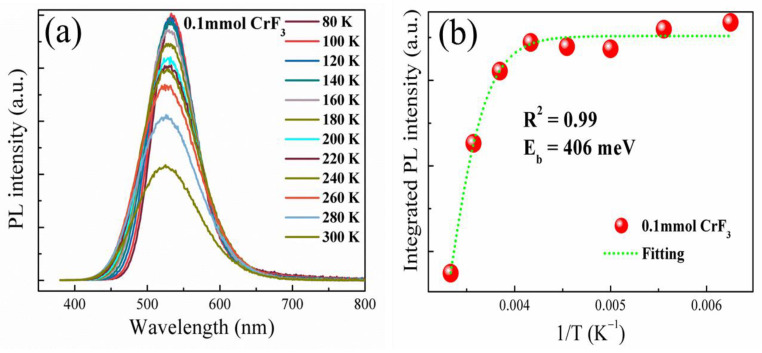
(**a**) Temperature-dependent PL spectra measured in the range of 80 to 300 K for the sample, synthesized with a CrF_3_ molar ratio of 0.1 mmol. (**b**) Integrated PL intensities measured for the corresponding sample at different temperatures (red solid symbols). Also shown is the fitting of the corresponding experimental data (green dashed curve) of the corresponding sample.

For a more profound understanding of the enhanced PL characteristics, we examined the temperature-dependent PL spectra of the Cs_4_SnBr_6−x_F_x_ sample, synthesized with a CrF_3_ molar ratio of 0.1 mmol, across a temperature range from 80 to 300 K. As illustrated in Figure 9, a temperature decrease triggers a substantial escalation in the PL intensity. It is worth noting that the PL QY of STEs is strongly influenced by the exciton binding energy. The thermal activation-mediated detrapping of STEs leads to a reduced rate of radiative recombination. The binding energy of STEs was derived by analyzing the integrated PL intensity (I_PL_) as a function of temperature using the Arrhenius equation [18]:

(1) $${\;I}_{\mathrm{PL}\;}\left(T\right)=\frac{I_{\mathrm{PL}\;}\left(T_{0}\right)}{1+\beta \exp\left(-E_{b}/k_{B}T\right)}$$

In this equation, I_PL_(T_0_) signifies the I_PL_ at 80 K, β symbolizes a constant associated with the density of centers involved in radiative recombination processes, k_B_ denotes Boltzmann’s constant, and E_b_ represents the exciton binding energy. Employing the Arrhenius equation to fit the experimental data, we ascertain the exciton binding energy E_b_ to be 406 meV for the sample synthesized with a CrF_3_ molar ratio of 0.1 mmol (refer to Figure 9b). It is remarkable that the E_b_ value for the Cr^3+^ doped sample substantially surpasses the 265 meV exhibited by the pure sample [18]. This suggests that thermal activation-induced detrapping of STEs has been effectively curtailed in the Cr^3+^ doped sample, culminating in a heightened emission from the STEs.

**Figure 10:**
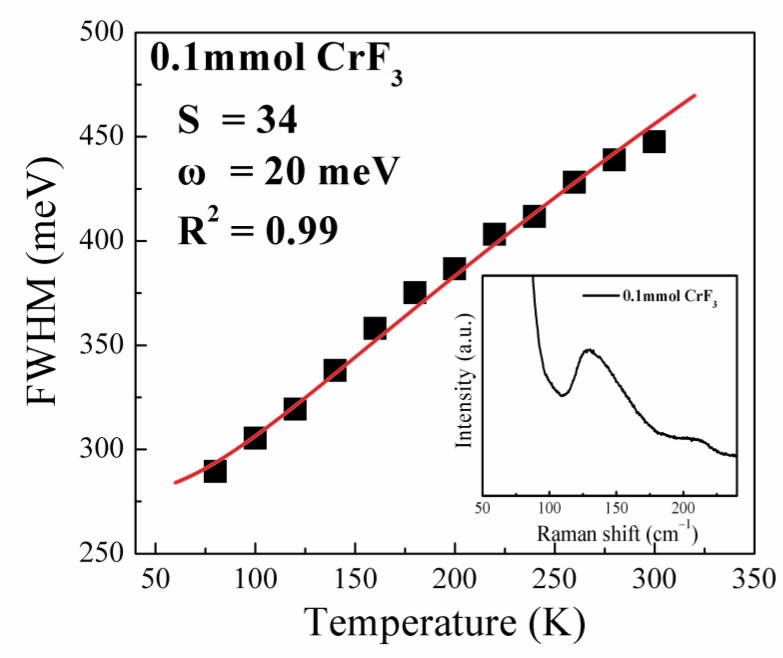
Temperature vs. PL linewidth observed for the sample, synthesized with a CrF_3_ molar ratio of 0.1 mmol, and the fitting of the experimental data (red line) using Equation (2). The insets in Figure 10 show the Raman spectrum of the corresponding sample.

From Figure 10, one can also see a remarkable reduction in the full width at half-maximum of the emission band with decreasing temperature for the sample prepared with a CrF_3_ molar ratio of 0.1 mmol. The breadth of this emission band is intrinsically linked to electron–phonon coupling and can be captured via the equation:

(2) $$FWHM\left(T\right)=2.36\sqrt{S}\hbar \omega \sqrt{\coth\frac{\hbar \omega }{2k_{B}T}}$$

Here, S denotes the Huang–Rhys factor, ℏω represents the phonon mode energy, T is the temperature, and kB signifies Boltzmann’s constant. By employing Equation (2) to fit the temperature-dependent FWHM of the PL peaks, we can extract the Huang–Rhys factor S, a widely recognized parameter for encapsulating the exciton–phonon coupling [18]. For the sample fashioned with a CrF_3_ molar ratio of 0.1 mmol, the S value was found to be as high as 34, as evidenced in Figure 10. The fitted data within Figure 10 reveal an optical phonon energy (E_LO_) of 20 meV (160 cm^−1^), aligning favorably with the Sn-Br stretching vibrational mode situated near 150 cm^−1^ in Cs_4_SnBr_6_ (refer to the inset of Figure 10) [24]. This confirms the engagement of a primary phonon mode, associated with the Sn-Br stretching vibrational mode near 150 cm^−1^, in the electron−phonon coupling. Consequently, we posit that this robust electron–phonon coupling amplifies the probability of STEs. Hence, based on these analyses, we infer that the robust electron–phonon coupling, in conjunction with the amplified exciton binding energy provoked by the CrF_3_ addition, is responsible for the intensified STE emission observed in the sample prepared with a CrF_3_ molar ratio of 0.1 mmol. It is noteworthy that the substantial substitution of Sn^2+^ by Cr^3+^ and Br^−^ by F^−^ could lead to significant distortion of Cs_4_SnBr_6_ structure, potentially creating additional nonradiative recombination centers. This might explain the observed decrease in PL intensity when the molar ratio of CrF_3_ exceeds 0.1 mmol.

**Figure 11:**
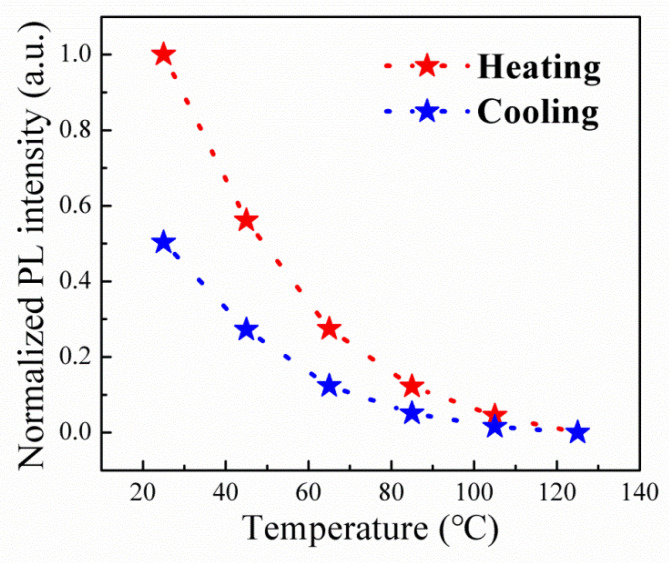
Heating and cooling cycling measurements of the Cr^3+^-doped sample prepared with a CrF_3_ molar ratio of 0.1 mmol at various temperatures.

To scrutinize the thermal resilience of the Cr^3+^-doped samples, the integrated PL intensities, as a function of temperature, were systematically tracked through successive heating and cooling cycles. As portrayed in Figure 11, an observable thermal quenching of PL is experienced by the Cr^3+^-doped sample as the temperatures escalated from 25 to 165 °C. Following these heating and cooling cycles, the PL intensity exhibits a reduction of approximately 50%. In stark contrast, the pure Cs_4_SnBr_6_ perovskite underwent a PL intensity reduction exceeding 90% post the identical thermal cycles [18]. Evidently, the Cr^3+^-doped Cs_4_SnBr_6−x_F_x_ perovskite manifests enhanced thermal and structural sturdiness in comparison to its pure Cs_4_SnBr_6_ perovskite counterpart.

## 4. Conclusions

In conclusion, Cr^3+^-doped Cs_4_SnBr_6−x_F_x_ perovskites were successfully fabricated employing the water-assisted wet ball-milling approach. The influence of CrF_3_ addition on the PL characteristics of Cs_4_SnBr_6−x_F_x_ perovskites was thoroughly examined. Findings revealed that these Cr^3+^-doped Cs_4_SnBr_6−x_F_x_ perovskites demonstrated a broad emission band, an appreciable Stokes shift, and robust green light emission, centered around 525 nm at room temperature, yielding a PL QY as high as 56.3%. Moreover, the Cr^3+^-doped Cs_4_SnBr_6−x_F_x_ perovskites manifested superior thermal stability when compared to their undoped equivalents. Based on the comprehensive analysis of photoluminescence measurements, the increased PL QY is suggested to originate from the augmented exciton binding energy of STEs and the suitable electron−phonon coupling brought about by the substantial distortion of the [SnBr_6_]^4−^ octahedra driven by the addition of CrF_3_.

## Data Availability

The data supporting the findings of this study are currently unavailable to the public but can be acquired from the authors upon reasonable request.

## Supplementary Materials

The following supporting information can be downloaded at: https://www.mdpi.com/article/10.3390/ma16186309/s1, PL QY calculation: The PL QY calculation formula and the values of the numerical integrals.
