# Supplementary material for: Effect of CrF3 Addition on Photoluminescence Properties of Lead-Free Cs4SnBr6−xFx Zero-Dimensional Perovskite
Source: Materials (Basel). 2023 Sep 20;16(18):6309. doi: 10.3390/ma16186309 (PMC10532708; doi:10.3390/ma16186309)
Supplement: Supplementary file 1 [file materials-16-06309-s001.zip › materials-2559610-supplementary.pdf]

Effect of CrF_3 Addition on Photoluminescence Properties of Lead-Free $Cs_4SnBr_{6-x}F_x$ Zero-Dimensional Perovskite

Jianni Chen, Haixia Wu, Yaqian Huang, Jisheng Xu, Xinye Lu, Wendi Zhou, Jie Song and Rui Huang *

School of Materials Science and Engineering, Hanshan Normal University, Chaozhou 521041, China
* Correspondence: rhuang@hstc.edu.cn

The PL QY calculation formula and the values of the numerical integrals

The Photoluminescent quantum yield (PL QY) of the Cr^{3+}-doped sample synthesized with a CrF_3 molar ratio of 0.1 mmol is calculated using the ratio of the number of photons emitted to the number of photons absorbed ($Q = N_{em}/N_{abs}$). Here, the number of emitted photons (N_{em}) is determined by the area under the spectrally corrected emission peak of the Cr^{3+}-doped sample ($N_{em}=A_{em\ sample}- A_{em\ ref}$). The value of N_{em} in our case is 1108215. Simultaneously, the number of absorbed photons (N_{abs}) is given by the difference in areas under the Rayleigh scattering peaks of a reference sample and the Cr^{3+}-doped sample under investigation ($N_{abs} = A_{scat\ ref} - A_{scat\ sample}$). The value of N_{abs} in our case is 1969100. Therefore, we can calculated the PL QY as following:

$$QY = \frac{N^{em}}{N^{abs}} = \frac{A^{em}_{sample} - A^{em}_{ref}}{A^{scat}_{ref} - A^{scat}_{sample}}$$

$$= \frac{1108215}{1969100} \approx 56.3\%$$
